# Supplementary figures and images for: Exploring the diagnostic potential of adding T2 dependence in diffusion-weighted MR imaging of the prostate
Source: PLoS One. 2021 May 27;16(5):e0252387. doi: 10.1371/journal.pone.0252387 (PMC8158951; doi:10.1371/journal.pone.0252387)

# S1 Fig. Patient and ROI selection

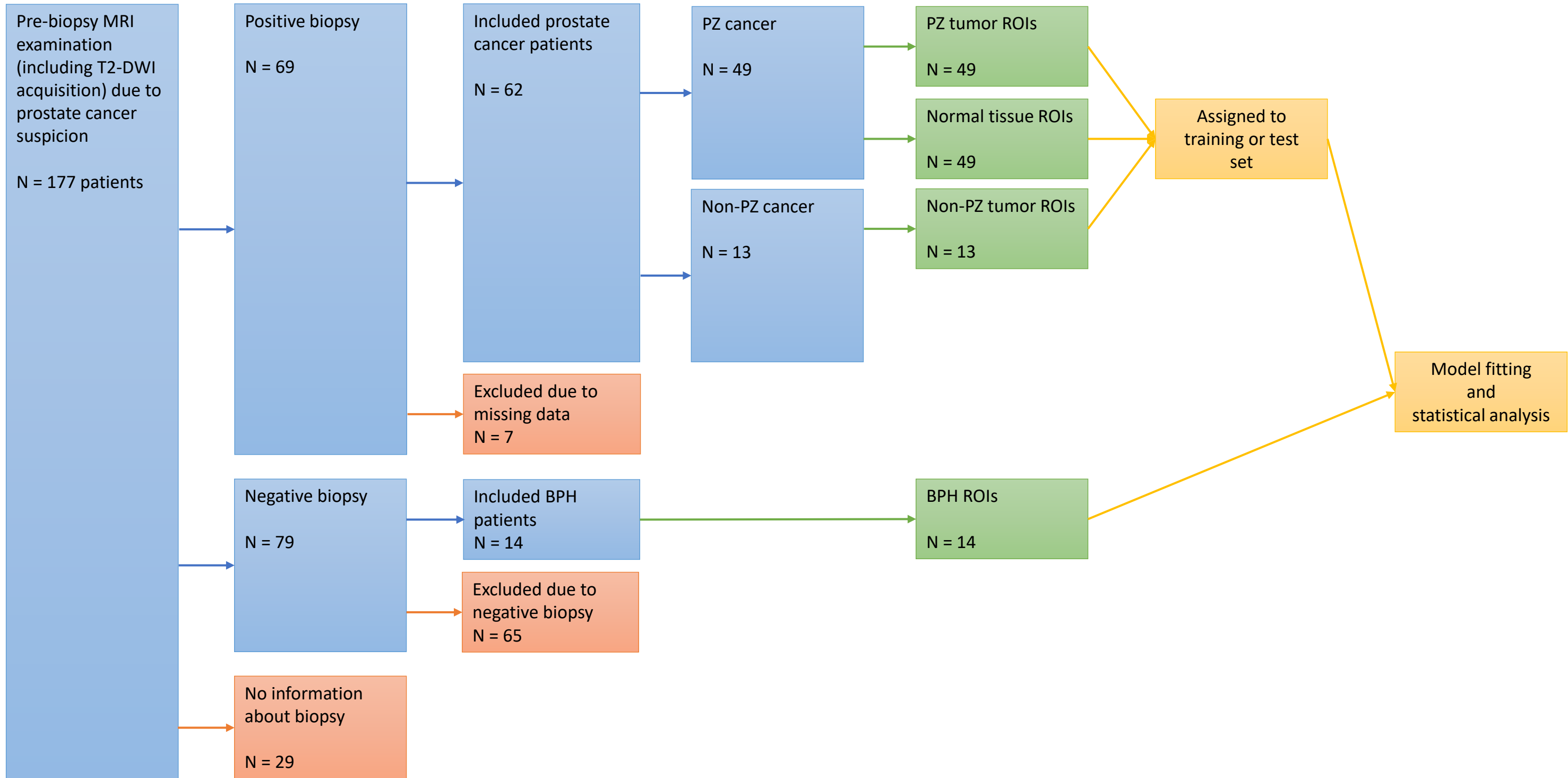

Supplement: S1 Fig — (PDF) [file pone.0252387.s002.pdf]
