# Supplementary material for: Exploring the diagnostic potential of adding T2 dependence in diffusion-weighted MR imaging of the prostate
Source: PLoS One. 2021 May 27;16(5):e0252387. doi: 10.1371/journal.pone.0252387 (PMC8158951; doi:10.1371/journal.pone.0252387)
Supplement: S2 Fig — SFslow (from the two-component (TC) and bi-exponential (BE) models) maps and corresponding ADC map are shown for a PZ tumor patient, a non-PZ tumor patient and a BPH patient, respectively. For the tumor patients, histology slides are also shown for comparison, while a corresponding T2-weighted image is shown in the BPH case as histology was not available for this patient. The lesions are denoted on the ADC maps with red arrows. On the histology slides, the tumors are denoted with dotted lines. (DOCX) [file pone.0252387.s003.docx]

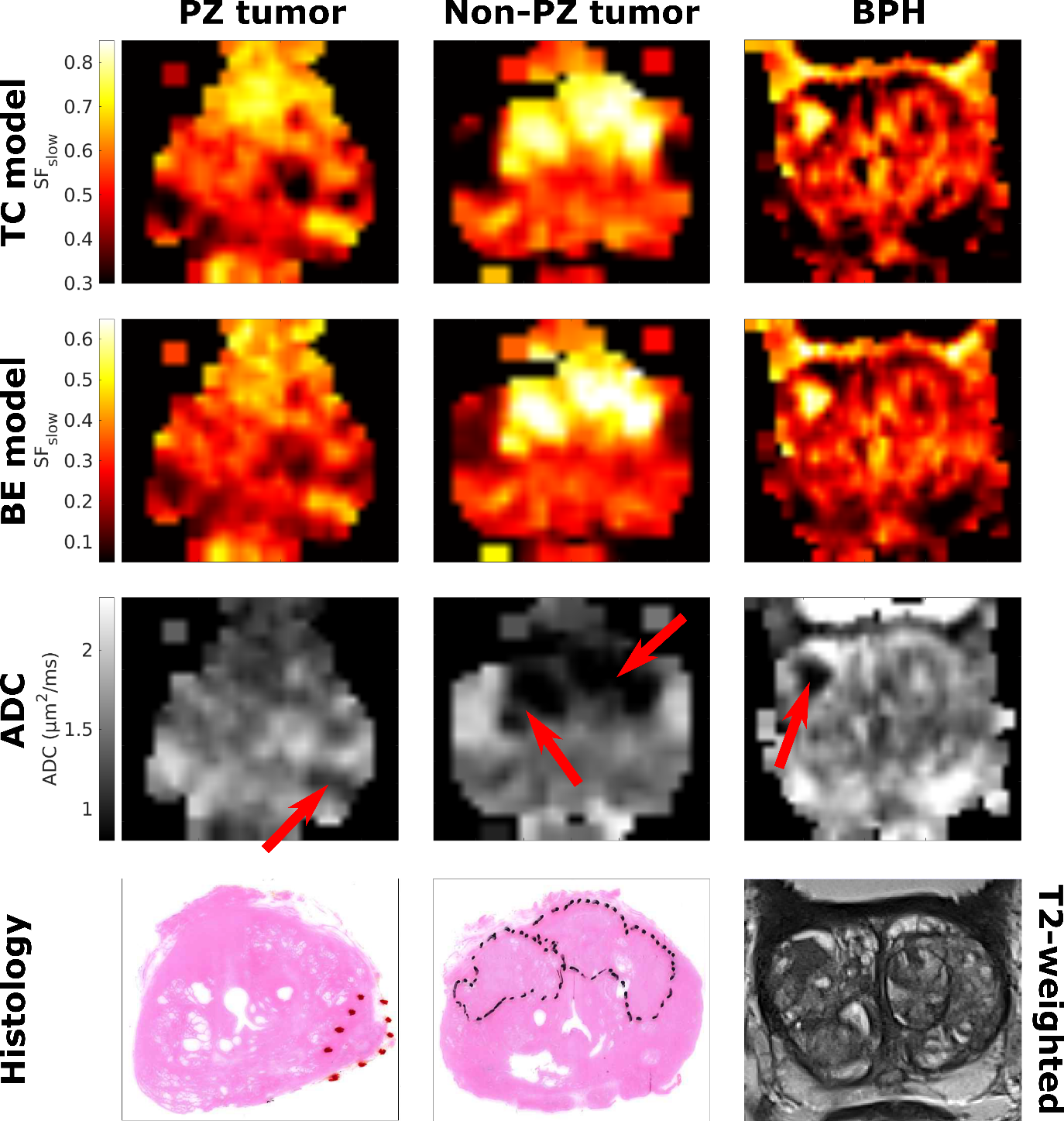


**S2 Fig. Examples of estimated value maps for three different patients.** SF_slow_ (from the two-component (TC) and bi-exponential (BE) models) maps and corresponding ADC map are shown for a PZ tumor patient, a non-PZ tumor patient and a BPH patient, respectively. For the tumor patients, histology slides are also shown for comparison, while a corresponding T2-weighted image is shown in the BPH case as histology was not available for this patient. The lesions are denoted on the ADC maps with red arrows. On the histology slides, the tumors are denoted with dotted lines.
