# Supplementary material for: Exploring the diagnostic potential of adding T2 dependence in diffusion-weighted MR imaging of the prostate
Source: PLoS One. 2021 May 27;16(5):e0252387. doi: 10.1371/journal.pone.0252387 (PMC8158951; doi:10.1371/journal.pone.0252387)
Supplement: S1 Appendix — (DOCX) [file pone.0252387.s004.docx]

S1 Appendix

## **Supplementary methods**

# Alternative models

To evaluate the individual effects of ADC and T2 on the two-component model, we extended the analysis and compared the results with signal fractions from three alternative models.

First, to investigate the effect of globally optimizing the T2 values, we fitted the two-component model,

$$\begin{aligned} \frac{SI}{{SI}_{0}}={SF}_{slow}\exp\left( -\frac{TE}{{T2}_{slow}} \right)\exp\left( -b*{ADC}_{slow} \right)+{SF}_{fast} exp\left( -\frac{TE}{{T2}_{fast}} \right)\exp\left( -b*{ADC}_{fast} \right), \#( SEQ Equation \backslash* ARABIC 1) \end{aligned}$$

with no constraints on the T2 values. Also here, we set SF_slow_+SF_fast_=1, ADC_slow_=0.3 µm^2^/ms and ADC_fast_=2.6 µm^2^/ms (19). This leaves four free parameters (SI_0_, SF_slow_, T2_slow_, T2_fast_) to be fitted to the four measurements.

The second one was an ADC-dependent bi-exponential model, but at TE=55 ms:

$$\begin{aligned} \frac{SI}{{SI}_{0}}={SF}_{slow} \exp\left( -b*{ADC}_{slow} \right)+{SF}_{fast}\exp\left( -b*{ADC}_{fast} \right),\#( SEQ Equation \backslash* ARABIC 2) \end{aligned}$$

where SF_slow_+SF_fast_=1, ADC_slow_=0.3 µm^2^/ms and ADC_fast_=2.6 µm^2^/ms as in the two-component model (19). SI_0_ and SF_slow_ were fitted to the two b-value measurements at TE=55 ms.

The third model was a T2-dependent bi-exponential model:

$$\begin{aligned} \frac{SI}{{SI}_{0}}={SF}_{slow}\exp\left( -\frac{TE}{{T2}_{slow}} \right)+{SF}_{fast} exp\left( -\frac{TE}{{T2}_{fast}} \right),\#\left( SEQ Equation \backslash* ARABIC 3 \right) \end{aligned}$$

with SF_slow_+SF_fast_=1, where we used the globally optimized T2_slow_ and T2_fast_ from the two-component model. SI_0_ and SF_slow_ were fitted to the two TE measurements at b=50 s/mm^2^ and b=700 s/mm^2^ separately.

# ADC and T2

For comparison, we also calculated the ADC at TE=55 ms, and T2 values at both b-values. Using

$$\begin{aligned} \frac{SI}{{SI}_{0}}=\exp\left( -b*ADC \right),\#\left( SEQ Equation \backslash* ARABIC 4 \right) \end{aligned}$$

SI_0_ and ADC were fitted to the two b-value measurements at TE=55 ms. Similarly, using

$$\begin{aligned} \frac{SI}{{SI}_{0}}=\exp\left( -\frac{TE}{T2} \right),\#\left( SEQ Equation \backslash* ARABIC 5 \right) \end{aligned}$$

SI_0_ and T2 were fitted to the two TE measurements at b=50 s/mm^2^ and b=70 s/mm^2^ separately.

Furthermore, the changes in ADC and T2 as a function of TE and b-value, respectively, were also calculated to see how they affect each other.

# Statistical analysis

The statistical analyses were divided into PZ and non-PZ tumors. Note that all PZ analyses were performed on the test set only, while the non-PZ analyses were carried out on all patients due to the low sample size.

The 9 metrics subject to statistical analyses were as following: SF_slow_ for the four alternative model calculations; ADC at TE=55 ms and T2 at both b-values; and the change in T2 values and ADCs as a function of b-value and TE, respectively. For the PZ analyses, the Wilcoxon signed-rank test was used to test for statistical significance between the tumor and normal tissue ROIs (n=24). For the non-PZ analyses, BPH ROIs from the BPH patients (n=14) were used for comparison with the non-PZ tumor ROIs (n=13), and the Mann-Whitney U test was used to test for statistical significance between these. All tests were two-sided. After a Bonferroni correction for 27 multiple comparisons (including the analyses in the main manuscript), *p*<0.0019 was considered statistically significant.

All results (including the metrics from the main manuscript) are shown in S1 Table.
