# Supplementary material for: Exploring the diagnostic potential of adding T2 dependence in diffusion-weighted MR imaging of the prostate
Source: PLoS One. 2021 May 27;16(5):e0252387. doi: 10.1371/journal.pone.0252387 (PMC8158951; doi:10.1371/journal.pone.0252387)
Supplement: S1 Table — CI = confidence interval, PZ = peripheral zone, BPH = benign prostatic hyperplasia, SF = signal fraction, ADC = apparent diffusion coefficient, TE = echo time. Note—For PZ tumors, tumor ROIs (n = 24) were compared with normal ROIs (n = 24), while for non-PZ tumors, tumor ROIs (n = 13) were compared with BPH ROIs (n = 14). Units are given in parentheses, except for SFslow which is unitless. A p-value<0.0019 was considered statistically significant, and the significant results are highlighted in bold. (DOCX) [file pone.0252387.s005.docx]

**S1 Table. Median [95% CI] of the mean region of interest (ROI) values calculated from the different models and parameters**

| Model | PZ tumors | | | Non-PZ tumors | | |
| --- | --- | --- | --- | --- | --- | --- |
|  | Tumor | Normal | p-value | Tumor | BPH | p-value |
| SF_slow_, T2-ADC two-component model | 0.77 [0.67, 0.79] | 0.32 [0.28, 0.36] | **< 0.0001** | 0.77 [0.73, 0.80] | 0.74 [0.68, 0.78] | 0.06 |
| SF_slow_, T2-ADC two-component model, no T2 constraints | 0.48 [0.44, 0.53] | 0.34 [0.29, 0.37] | **< 0.0001** | 0.47 [0.36, 0.60] | 0.47 [0.35, 0.56] | 0.58 |
| SF_slow_, ADC-dependent bi-exponential model, TE=55 ms | 0.54 [0.43, 0.58] | 0.17 [0.13, 0.20] | **< 0.0001** | 0.54 [0.46, 0.60] | 0.49 [0.46, 0.56] | 0.30 |
| SF_slow_, ADC-dependent bi-exponential model, TE=73 ms | 0.54 [0.43, 0.60] | 0.12 [0.10, 0.16] | **< 0.0001** | 0.57 [0.51, 0.60] | 0.51 [0.44, 0.59] | 0.09 |
| SF_slow_, T2-dependent bi-exponential model, b=50 s/mm^2^ | 0.68 [0.62, 0.73] | 0.14 [0.09, 0.31] | **< 0.0001** | 0.79 [0.45, 0.94] | 0.68 [0.51, 0.84] | 0.25 |
| SF_slow_, T2-dependent bi-exponential model, b=700 s/mm^2^ | 0.66 [0.59, 0.73] | 0.53 [0.47, 0.78] | 0.0164 | 0.70 [0.55, 0.92] | 0.66 [0.44, 0.76] | 0.58 |
| ADC, TE=55 ms (µm^2^/ms) | 0.94 [0.86, 1.14] | 1.86 [1.75, 1.98] | **< 0.0001** | 0.94 [0.85, 1.09] | 1.03 [0.91, 1.09] | 0.32 |
| ADC, TE=73 ms (µm^2^/ms) | 0.94 [0.84, 1.16] | 2.02 [1.88, 2.11] | **< 0.0001** | 0.89 [0.83, 1.00] | 0.99 [0.85, 1.12] | 0.09 |
| Change in ADC (%) | 0.81 [-5.14, 2.96] | 8.53 [5.47, 10.32] | **0.0002** | -6.17 [-8.53, 2.15] | -2.38 [-8.83, 3.35] | 0.72 |
| T2, b-value=50 s/mm^2^ (ms) | 81 [75, 90] | 247 [154, 302] | **0.0002** | 69 [48, 189] | 81 [61, 113] | 0.40 |
| T2, b-value=700 s/mm^2^ (ms) | 86 [74, 106] | 127 [72, 159] | 0.0093 | 111 [51, 166] | 85 [75, 148] | 0.68 |
| Change in T2 (%) | 1 [-6, 30] | -50 [-53, -38] | **0.0001** | 17 [1, 66] | 13 [-6, 44] | 0.58 |

CI = confidence interval, PZ = peripheral zone, BPH = benign prostatic hyperplasia, SF = signal fraction, ADC = apparent diffusion coefficient, TE = echo time.
Note—For PZ tumors, tumor ROIs (n=24) were compared with normal ROIs (n=24), while for non-PZ tumors, tumor ROIs (n=13) were compared with BPH ROIs (n=14). Units are given in parentheses, except for SF_slow_ which is unitless. A p-value<0.0019 was considered statistically significant, and the significant results are highlighted in bold.
